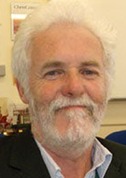# Methanol Synthesis from CO_2_ Hydrogenation

**DOI:** 10.1002/cctc.201900401

**Published:** 2019-07-10

**Authors:** Michael Bowker

**Affiliations:** ^1^ Cardiff Catalysis Institute School of Chemistry Cardiff University Cardiff CF10 3AT UK; ^2^ UK Catalysis Hub Research Complex at Harwell(RCaH) Rutherford Appleton Laboratory Harwell Oxon OX110FA UK

**Keywords:** Carbon dioxide hydrogenation, copper catalysts, methanol plant, sustainable methanol, ZnO catalysts

## Abstract

In the future we will be phasing out the use of fossil fuels in favour of more sustainable forms of energy, especially solar derived forms such as hydroelectric, wind and photovoltaic. However, due to the variable nature of the latter sources which depend on time of day, and season of the year, we also need to have a way of storing such energy at peak production times for use in times of low production. One way to do this is to convert such energy into chemical energy, and the principal way considered at present is the production of hydrogen. Although this may be achieved directly in the future via photocatalytic water splitting, at present it is electrolytic production which dominates thinking. In turn, it may well be important to store this hydrogen in an energy dense liquid form such as methanol or ammonia. In this brief review it is emphasised that CO_2_ is the microscopic carbon source for current industrial methanol synthesis, operating through the surface formate intermediate, although when using CO in the feed, it is CO which is hydrogenated at the global scale. However, methanol can be produced from pure CO_2_ and hydrogen using conventional and novel types of catalysts. Examples of such processes, and of a demonstrator plant in construction, are given, which utilize CO_2_ (which would otherwise enter the atmosphere directly) and hydrogen which can be produced in a sustainable manner. This is a fast‐evolving area of science and new ideas and processes will be developed in the near future.

## Graphical abstract

**Methanol synthesis**: This Minireview describes the mechanism of methanol synthesis, and considers a way of synthesizing methanol from sustainable, non‐fossil hydrogen. This combines electrolysis of water using solar energy, combined with CO_2_ garnered from a coal power plant. It covers the synthesis of traditional CZA (Cu−Zn−Al) based catalysts and the possibilities for novel materials.

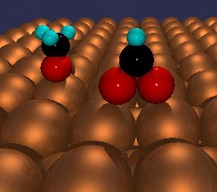

## Introduction

Methanol is also known as ‘wood’ alcohol because it was originally produced from the pyrolysis of wood, though it is believed that it was only produced in a pure form by Robert Boyle in 1661.^1^ It is a very valuable intermediate chemical, yielding products such as formaldehyde, dimethyl ether, acetic acid, besides its direct use as a fuel.

It has been produced catalytically since the1920s when BASF invented a process operating at high temperature and pressure and using a variety of oxide catalysts, especially a mixed Cr_2_O_3_‐ZnO material.^2^ Much later a new process was developed by ICI.^3^, ^4^ This was a Cu/Zn/Al_2_O_3_ catalyst (hence CZA for short), operating at lower temperature and pressure and part of the reason it could be applied is that it was combined with a desulphurization unit up front, since Cu is very sensitive to S poisoning, and it was associated with the switch from coal –based feedstocks to natural gas. The first commercial catalysts was ICI 51‐1 and the first plant using it operating in 1966. This catalyst has remained essentially the same over the succeeding years, but with improved sintering resistance and including some additives.^5^ The ICI business was taken over by Johnson Matthey and the catalyst developments are shown by the evolution of the initial 51‐1 catalyst (quickly followed by 51‐2) to 51‐9 and beyond.^6^ A number of other companies offer methanol synthesis catalysts, including Haldor Topsoe and BASF. The essential reaction is as follows, but this will be discussed in more detail below [Equation (1)]:

(1) $$\mathrm{CO}+2H{}_{2}\to \mathrm{CH}{}_{3}\mathrm{OH}\left(\Delta H{}_{r}{}^{0}=-91\;\mathrm{kJ}\;\mathrm{mol}{}^{-1}\;\Delta S{}_{r}{}^{0}=\;-220\;J\;K{}^{-1}\;\mathrm{mol}{}^{-1}\right){}^{7}$$

With our increased understanding of climate change we are now very aware that our use of fossil fuels has disrupted the near steady state behaviour of the atmosphere of the last 10000 years, where the evidence is that the CO2 level was 240 ppm (±40 ppm, the extremes being between glacial and interglacial periods: note we are currently in an interglacial period), whereas it has now passed 400 ppm, which may be the highest level for 20 M years.^8^ This is starkly illustrated in a movie from Ed Hawkins at the University of Reading,^9^, ^10^ which shows the month‐by‐month trends in CO_2_ increase 1850–2016, in terms of global average temperatures: these had increased by ∼1.5 °C to that point. Hence there is an urgent need for processes which do not use fossil fuels. Current methanol production is at the level of >20Mt/a, but it is almost entirely from fossil fuel sources, and mainly from natural gas. This then also involves a double whammy for the atmosphere because methanol steam reforming to produce the CO_2_/CO/H_2_ mix required for traditional methanol synthesis is highly endothermic and requires a massive energy input. The reactor tubes are radiatively heated externally by the combustion of natural gas.

And so there has been a big effort in recent years for a direct method of methanol production without the use of fossil fuels, but utilizing CO_2_ already in the environment, or trapped before it reaches the environment, combined with hydrogen produced from renewable energy. This reaction, then, is rather different from the one above, with somewhat different thermodynamics, though it is still exothermic [Equations (2) and (2)]:

(2) $$\mathrm{CO}{}_{2}+3H{}_{2}\to \mathrm{CH}{}_{3}\mathrm{OH}+H{}_{2}O\left(\Delta H{}_{r}{}^{0}=-50\;\mathrm{kJ}\;\mathrm{mol}{}^{-1};\;\Delta S{}_{r}{}^{0}=\;-175\;J\;K{}^{-1}\;\mathrm{mol}{}^{-1}\right){}^{7}$$

Important in this respect is also the likelihood of the reverse water‐gas shift reaction (RWGS) occurring at the same time, which is in contrast to the previous two reactions, endothermic

(3) $$\mathrm{CO}{}_{2}+H{}_{2}\to \mathrm{CO}+H{}_{2}O\left(\Delta H{}_{r}{}^{0}=39\;\mathrm{kJ}\;\mathrm{mol}{}^{-1},\Delta S{}_{r}{}^{0}=\;42\;J\;K{}^{-1}\;\mathrm{mol}{}^{-1}\right){}^{7}$$

This review concerns what is often called ‘green’ methanol synthesis. It is called that because it results in no new net CO_2_ emissions to the atmosphere, and can potentially reduce CO_2_ emissions because the renewable energy in the H components of the molecule represent around half of its heat of combustion. How ‘green’ it is, of course depends on a set of factors, not least being the enormous cost of electrolysers used to produce the hydrogen from water splitting, both economic and environmental. However, to offset that, there is no need for the steam reformer which is normally used and which itself has enormous environmental and economic costs.

## Conventional Process

**Figure 1:**
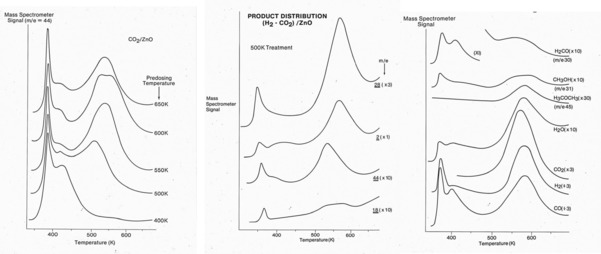
Temperature programmed desorption (TPD) experiments after dosing gases at various temperatures on ZnO powder, and cooling in the gas to ambient. (Left panel) CO_2_ TPD showing the activated nature of adsorption into the most stable state desorbing at 550 K; (middle panel) after adsorption of a mixture of CO_2_ and H_2_ at 500 K and cooling, showing mainly CO and H_2_ production, but also with CO_2_ and H_2_O; (right panel) products after the adsorption of H_2_CO at 500 K, with cooling. The product distribution is very similar to that seen after methanol dosing, and shows a variety of products evolving near‐coincidently with the formate decomposition, including formaldehyde itself, dimethyl ether, and methanol.^11^, ^12^. Adapted from ref 11,with thanks to the Royal Society of Chemistry.

**Figure 2:**
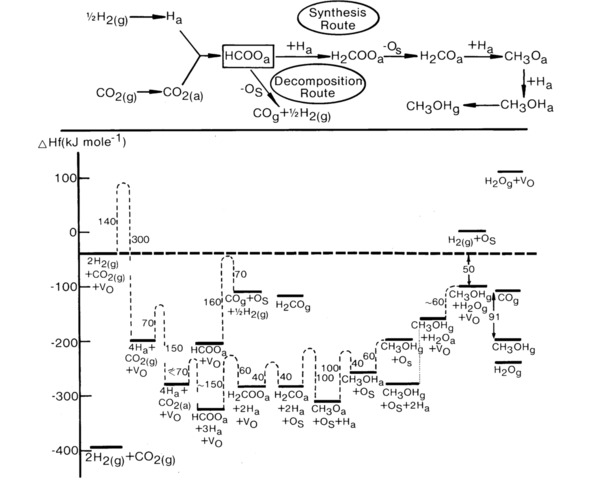
The methanol synthesis mechanism and kinetics for the reaction on ZnO.^12^

Work in ICI in the early days investigated the details of the mechanism and kinetics of the reaction.^11^, ^12^, ^13^, ^14^, ^15^ Beginning with the mechanism on ZnO powder alone,^11^, ^12^ it was found by TPD that CO_2_ can adsorb strongly on the surface, especially if adsorbed above 400 K and it can co‐adsorb with H_2_ in a mixed gas phase to produce an intermediate, see Figure 1. The evidence of an intermediate is the desorption of several products, mainly CO and H_2_, at a coincidently at 570 K. In contrast there is no significant adsorption of CO, concomitant with its low heat of adsorption of ∼60 kJ mol^−1^. The same kind of desorption was found from methanol or formaldehyde adsorption and so the inference was that a common intermediate was a crucial one for methanol synthesis and was likely to be the most abundant surface intermediate during synthesis on ZnO. Furthermore it follows that CO_2_ is the microscopic C source for methanol. As a result of all this work a mechanism for the reaction on ZnO was evolved (Figure 2).^11^, ^12^ Here the formate is identified as the common intermediate, and the most stable intermediate in these studies, as seen in the TPD, and more recent mechanisms are generally similar to this one, for example, the work of Medford et al.^16^ Note that both formaldehyde and methanol are seen to evolve, albeit at low level, with the formate decomposition, showing that the formate hydrogenation is the rate determining step in methanol synthesis.

The mechanism involves CO_2_ as the microscopic source of carbon in methanol, on the left side of Figure 2, at anion vacancies in the ZnO lattice. Hydrogen is dissociated and reacts with adsorbed CO_2_ to make the formate intermediate. The formate decomposition route mainly involves re‐oxidation of anion vacancies which are seen as important in this scheme and as a way of adsorbing CO_2_ onto the surface, perhaps by adsorption of an oxygen from the CO_2_ into an anion vacancy, with electron pick up into the molecule from the vacancy polaron. Then sequential hydrogenation of the molecule occurs to form the dioxymethane intermediate, which then loses an oxygen to the anion vacancy to fill it (labelled O_s_ in Figure 2). Further hydrogenation then takes place to make adsorbed methanol, which is then lost to the gas phase. The anion vacancy is then re‐formed by water production.

**Figure 3:**
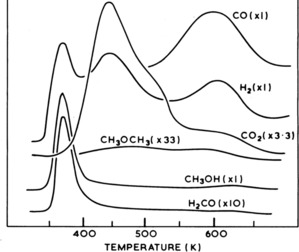
Desorption products after exposure of a Cu/ZnO/Al_2_O_3_ catalyst to methanol at ambient temperature. a) dimethyl ether (45 amu x33), b) hydrogen (2 amu x1), c) CO_2_(44 amu x3.3), d) methanol (29 amu), e) formaldehyde (31 amu x10), f) CO (28 amu x1).

These findings for ZnO were essentially confirmed to be the case for Cu based catalysts too,^15^ and were also inferred from surface science studies of the reactivity of well‐defined Cu surfaces.^12^, ^18^, ^19^, ^20^, ^21^, ^22^, ^23^ It was shown in studies by Madix et al and Bowker et al that methanol oxidation on well‐defined single crystal surfaces of Cu produced methoxy and formate intermediates on the surface. The formate decomposes on copper characteristically to produce coincident evolution of CO_2_ and H_2_. The TPD from a CZA catalyst is shown in Figure 3 and also shows a formate intermediate, with coincident CO_2_ and H_2_ desorption, as for copper single crystals, but at lower temperature (∼500 K) than for ZnO, showing its lower stability on Cu. The ZnO formate, yielding CO, is also seen at ∼600 K. The crucial role of CO_2_ as the carbon source in methanol synthesis was later confirmed with reactor measurements, including those utilizing labelled CO_2_ 14, 15, 17

As a result of all this work the mechanism of the reaction was concluded to be the following [Equations (3) and (3)]

(3) $$\mathrm{CO}{}_{2}+2H{}_{2}\to \mathrm{CH}{}_{3}\mathrm{OH}+O{}_{a}$$

**Figure 4:**
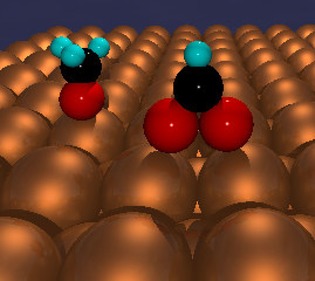
Ball models of the surface methoxy (left) and formate species (right) on a metal surface; red‐oxygen, blue‐hydrogen, black‐carbon and gold, the metal.

That is, CO_2_ is directly hydrogenated to methanol on the surface, via hydrogenation of the formate intermediate, but this leaves an adsorbed oxygen atom (O_a_). A model of the formate (and methoxy) intermediate is shown in Figure 4, with the formate being bidentate, as observed in many studies, but which may become monodentate during the hydrogenation step. For steady state reaction, obviously the oxygen has to be removed again and that occurs by reaction with CO adsorbing from the gas phase

(4) $$\mathrm{CO}+O{}_{a}\to \mathrm{CO}{}_{2}$$

So the overall reaction is a combination of these two reactions, and produces little water in the exit stream^4^

(1) $$\mathrm{CO}+2H{}_{2}\to \mathrm{CH}{}_{3}\mathrm{OH}$$

There has now been an enormous amount of work on the mechanism and kinetics of the reaction, with a variety of models to explain the particular efficacy of this system. These include the importance of Cu^+^ sites in the ZnO lattice for the reaction, originally proposed by Klier,^24^, ^25^ the formation of brass (CuZn alloy) in the catalyst,^26^, ^27^ the formation of surface Zn on Cu (a kind of SMSI effect),^26^‐^29^ and Zn at the interface plane edges of the Cu catalyst,^30^ to name just some of them. So, the exact nature of the active site still remains somewhat unclear, but almost certainly involves some kind of close coupling in the mechanism between the Cu nanoparticles and the support oxide, with the latter likely to be the anchor point for one of the oxygen atoms in CO_2_.

However, in relation to this, there is a general observation that the activity for methanol synthesis is generally linearly related to the Cu metal surface area, implying that Cu is involved in the crucial rate determining step (RDS). The ICI workers used reactive frontal chromatography to titrate surface sites by N_2_O decomposition^31^, ^32^ (N_2_O+*→N_2_+Oa), which stops when the surface layer is saturated. This has become something of a standard method for determining the Cu surface area, notwithstanding the obvious fact that the surface ratio O : Cu varies with crystal plane exposed! The nature of the Cu nanoparticles is undoubtedly changed somewhat, depending on the support material to which it's attached, possibly due to electron density changes at the Fermi level. Such changes would then affect the stability of the formate species and its hydrogenation to methanol.

We must remember that the original invention of the CZA catalyst has been a tremendous success, evidenced by the fact that, over the last 50 years, the essential composition has changed relatively little. However, we must also note that when considering ‘green’ methanol synthesis (equation 2), then there is a high level of water at the reactor exit, and the conventional catalyst may show activity decline in hydrous conditions at high temperature and pressure.^33^ However, using a silica support may improve the situation^33^ and the Dalian group recently reported a catalyst based on ZnO as the active phase, that may have improved properties for the reaction.^34^

## ‘Sustainable’ Methanol Synthesis

If we are to move away from fossil‐fuel based methanol production, then we need to use a ‘green’ source of carbon. Hence we need to use CO, or (more likely) CO_2_, from sources which would otherwise liberate it directly to the atmosphere, or it could be taken directly from the atmosphere itself. Also the H_2_ must not be derived from fossil fuels.

There is a further environmental aspect to this, and that concerns hydrogen and the potential hydrogen economy. When it comes to the use of methanol as a strategic fuel or chemical intermediate both the C and H have to be sourced sustainably, but if the hydrogen is a source of energy, then we may need to store it in a more energy‐dense form and there are a number of options for that. We may wish to store it as ammonia, or methane, or indeed, as methanol itself. Whichever of these is run, the N or C is merely the carrier for the (hopefully) green hydrogen, which represents a stored form of solar energy (remembering that wind power is a form of solar power generated from global temperature fluctuations).

For sustainable methanol production, then, we may need to carry out reaction 2, with co‐production of water. The hydrogen can be sourced from renewable electricity by electrolysis, while the CO_2_ can be sourced from ammonia plants, coal‐fired power stations, cement works and steel works, the latter which also has CO, or from CCS units, or could be taken directly from the air itself.

## Demonstrator Plants for Sustainable Methanol Synthesis

**Figure 5:**
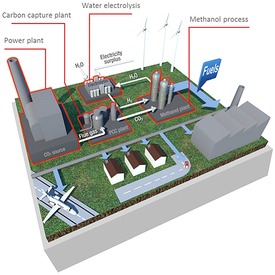
Schematic of the 500 t/y mefCO2 plant built at Niederaussem to convert CO_2_ from the power plant to methanol using hydrogen from solar‐generated electricity via electrolysis. Courtesy of Angel Sanchez Diaz at iDeals, Madrid.

There are a number of demonstrator plants for this technology, but the one I will mainly describe is one with which my group is involved, namely the pan‐European mefCO2 project.^35^ A schematic figure of the plant is shown in Figure 5 below, and it has been constructed very recently at Niederaussem near Cologne, at the RWE coal‐fired power station, as the source for CO_2_. It is capable of producing 500 t/y of methanol. It consists of a PEM based electrolyser,^36^ which produces valuable medical grade oxygen as well as the required hydrogen. The CO_2_ is then cleaned in an amine scrubber system,^37^ to remove many of the impurities from the stream, especially S and N compounds, before feeding into the plant. The two reactants are pressurized before feeding into the reactor, which operates at around 500 K and high pressure. The methanol is separated at the exit stream and exported, while unused reactants are fed, via a recycle compressor, back to the front of the reactor, where the feed is then topped‐up with fresh reactant.

There are now a number of other such projects around the world, notably the plant run for some time in Iceland by Carbon Recycling International (CRI) utilising geothermal energy, and a much bigger plant commissioned for China.^38^

## The Catalyst for Methanol Synthesis

**Figure 6:**
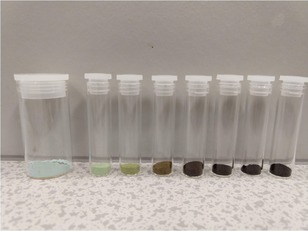
CZA catalyst powder after synthesis and after drying at 110 °C (left sample) and (right) after calcination from 200 to 500 °C, every 50 °C. Catalyst preparation and photo courtesy of Dr James Hayward, Cardiff Catalysis Institute, Cardiff University.

The catalyst of choice for industry is the CZA (Cu, ZnO, Al_2_O_3_) material. This is generally made by co‐precipitation of the nitrates of the three cation components, using a base, usually sodium carbonate, at a temperature of ∼70 °C and with slightly alkaline conditions at the end of the procedure. It is then washed (e. g. to remove Na ions) and dried at ∼120 °C. At this stage the catalyst is light blue in colour, Figure 6. The catalyst is then dried in a rotary drier and calcined, usually to ∼350 °C after which it is black as the oxides are formed, Figure 6. The catalyst is then mixed with binders and lubricants (e. g. graphite) for extrusion to form cylindrical pellets of ∼5mm cross‐section after firing. The catalyst can then be sold as is, or in a pre‐reduced and passivated form. Like most metallic catalysts, the reduced material can be pyrophoric if not handled in the correct way. The catalyst is then fed into the reactor carefully and the plant start‐up involves first slow reduction of the catalyst, under carefully temperature‐controlled conditions, before finally running at temperature in the reacting gases.

The catalyst has evolved over the period since its invention and application in the 1960s, mainly to improve longevity by the addition of components such as Mg, but it is otherwise essentially the same as it ever was.

Of course, ‘green’ methanol synthesis is not standard methanol synthesis, proceeding according to reaction 2 rather than 1. This then means that there is a significant water level at the outlet of the reactor bed, which in turn has implications for decreased catalyst activity and lifetime.^33^ This may require further modifications to catalyst structure/composition for the improvement of performance.

## Equilibrium and Plant Design

**Figure 7:**
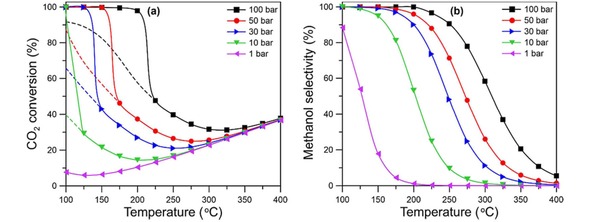
Equilibrium methanol conversion and selectivity for a CO_2_/H_2_ mix with a ratio 1/3, and its dependence on pressure and temperature, the dashed lines represent gas phase equilibrium.^40^ Reprinted with permission from the American Chemical Society.

Methanol synthesis runs near equilibrium, since it is an exothermic reaction, and yield diminishes at high temperatures (see Figure 7).

**Figure 8:**
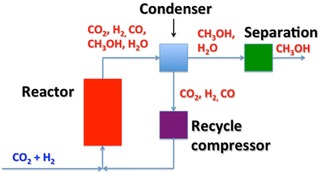
A simplified diagram of the main components of a ‘green’ methanol synthesis plant.

So the thermodynamics dictate for low temperature operation (and high pressure due the molar reduction in synthesis), but kinetics dictate for high temperature operation and a compromise is reached at ∼230 °C, where the equilibrium yield at 50 bar is ∼30 %, fig 7,^40^ and the rate is high with good selectivity. In turn, these various factors dictate reactor design. It must be capable of operating at high pressure, moderate temperature, but most importantly there must be 1) an efficient way to remove the exotherm from the reactor (overall methanol synthesis, so heat release) and 2) a recycle facility to send unreacted gases back to the front of the reactor after condensing out the methanol and water. Regarding item 1, the coolant can be water/steam or some other gas/fluid, and the heat recovered is fed back, via heat exchangers into the steam production for steam reforming in a normal plant. For a renewable methanol plant this heat will need to be used elsewhere, for instance, in preheat of the reactor gases, or distillation of the methanol, or exported for plant area/domestic use. Regarding item 2, if the yield is, say, 25 % then obviously the recycle ratio needs to be at least ∼5 times for efficient conversion of all the reacting gases, which have cost so much to produce in the first place. The recycle compressor represents a significant fraction of the capital cost of a reactor. A simplified diagram of such a plant is given in Figure 8

So the main differences with a ‘renewable’ methanol plant are

1. Clean hydrogen production from sustainable sources (probably wind‐generated electricity in northern Europe, solar in desert locations) via electrolysis.
2. Heat recovery. This is utilised very differently from a conventional plant and may have to be exported from the site (e. g. local domestic), depending on its level of integration within the plant itself or into other on‐site processes.
3. Methanol separation. This will be costly, since there will be a high level of water at the reactor exit, and may have to utilise novel technologies for most efficiency, for instance membrane technology.

## Novel Catalysts

It will be very difficult to get away from conventional catalysts for the process, especially since they already utilise ‘earth‐abundant’ materials (Cu, Zn, Al). However, there is a need for more water‐tolerant catalysts that can give longer lifetime in the plant. Nonetheless there are a number of academic attempts to make new kinds of catalysts that could work well under synthesis conditions. One example of this (though not economic or environmentally‐benign) is the use of Pd for methanol synthesis from CO_2_+H_2_. It would be imagined at first that Pd would be a poor catalyst for methanol synthesis, and indeed that is the case,^41^ since it preferentially catalyses the reverse water gas shift (RWGS) reaction to produce CO, equation 3 above, and also produces small, but not insignificant amounts of methane. However, if the Pd is alloyed with other metals then selectivity can be enhanced. Perhaps the simplest example of this is that if the Pd is supported on ZnO, then high temperature reduction (>∼300 °C) results in the formation of the 1 : 1 PdZn alloy,^41^, ^42^ which then shows very good selectivity to methanol.^41^, ^42^, ^43^, ^44^, ^45^ Such catalysts can be synthesised in a number of ways, and with other supports, such as ZnAl_2_O_3_, to improve performance, which can match well with that of conventional CZA catalysts. They can also be made to operate with Pd levels as low as 1 % and be effective, but they are still too expensive.

So then, of course, the search is on for other earth‐abundant materials significantly different from CZA. Since Pd shows promise, maybe a more economically viable option would be to go for a cheaper element in that group or nearby, such as Ni or Co. However, as is well known, Ni is a good methanator in a CO/CO_2_−H_2_ mix, and is the metal of choice for that reaction (and its reverse, the steam reforming of methane). However, when it is alloyed with other elements it can show some activity for methanol synthesis. So for example Studt et al^46^ have published on NiGa as a potential methanol synthesis catalyst, based upon theoretical considerations of the nature of the electronic structure of the material. However, that material was only tested at ambient pressure and to quote one of the authors of that paper “Ni−Ga catalysts for methanol synthesis cannot be considered for practical application at this stage… and cannot compete with CZA catalysts..”.^47^ Nevertheless, they do indeed make some methanol, and with reasonable selectivity. We have made and tested such materials at higher pressure and they are considerably inferior to CZA catalysts, with lower activity, some methane production, and requiring rather harsh conditions (∼700 °C) for pre‐reduction, and show a considerable decline in activity over time, as also shown by Sharafutdinov.^47^ More recently Singh et al reported CoGa as a possible catalyst.^48^ Other approaches involve confinement of Cu‐ZnO systems in restricted environments, such as in zeolites to imbue them with some acidic properties^49^ and thereby induce new reactivity, or by confinement in MOFs.^50^

Of course, as well as using novel materials, we can consider new ways of making Cu‐based catalysts. One approach, which could be environmentally beneficial is to avoid a solvent in the preparation and we have recently reported data for such a catalyst produced by anti‐solvent methods utilising supercritical CO_2_ 51 or gas phase chemical vapour impregnation (CVI).^41^, ^52^ This produces good catalysts with high dispersion of the Cu, but for efficiency, the former requires excellent recycling of the CO_2_. Likewise, sol immobilization methods can produce materials of rather homogeneous and small particle size.

We have recently reported that smaller Cu particle size (and hence, in principle, better activity, since activity is Cu surface area dependent) can be produced partly using two dimensional layered materials.^53^ Li et al subsequently reported a similar observation when they made Cu‐ZnO catalysts doped with Ga.^54^

## Conclusions

In the future we will be phasing out the use of fossil fuels in favour of more sustainable forms of energy, especially solar derived forms such as hydroelectric, wind and photovoltaic. However, due to the time‐variable nature of the latter sources we also need to have a way of storing such energy at peak production times for use in lean times. One way to do this is to convert such energy into chemical energy, and the principal way considered at present is the production of hydrogen. Although this may be achieved directly in the future via photocatalytic water splitting, at present it is electrolytic production which dominates thinking. In turn, it may well be important to store this hydrogen in an energy dense liquid form such as methanol or ammonia. In this brief review it is emphasised that CO_2_ is the microscopic carbon source for current industrial methanol synthesis, although when using CO in the feed, it is CO which is hydrogenated at the global scale. However, methanol can be produced from pure CO_2_ and hydrogen using conventional and novel types of catalysts. Examples of such processes and of a demonstrator plant in construction are given.

## Conflict of interest

The authors declare no conflict of interest.

## Biographical Information

***Prof. Michael Bowker** completed his PhD in Surface Science in Liverpool (UK) under the supervision of Prof. David King in 1977. After two years as a Research Fellow at Stanford University (USA) he returned to the UK as a Senior Research Scientist for the ICI corporate laboratory in 1979. In 1987, his academic career kicked off again as Founding Assistant Director of the Leverhulme Centre for Innovative Catalysis at the University of Liverpool, swiftly followed, as a founding member, of the Interdisciplinary Research Centre in Surface Science at the same university. In 1993 he was hired as Head of Physical Chemistry at Reading University where he remained for 10 years. Since his move to Cardiff University in 2003, he has held many roles including, Professor of Surface Chemistry, Head of Heterogeneous Catalysis and Surfaces Group, Founder of the Wolfson Nanoscience Laboratory (2006), Deputy Director of the Cardiff Catalysis Institute (2009). His main current interests are CO_2_ conversion, selective oxidation, magnetocatalysis and photocatalysis for hydrogen production*.